# Dysentery Treated by Hydrochloride of Emetin

**Published:** 1914-06

**Authors:** 


**Dysentery Treated by Hydrochloride of Emetin.** Juan Carlos Labat, La Semana Medica, August 4, 1913, reports the use of emetin in a case of dysentery which had been lasting 7 years and which had defied all treatment. During the last five months the patient had 20 bowel movements a day—blood and mucus—and was greatly emaciated. Then 0.05 grammes of emetin were injected. The day following, the patient had 9 movements. The next day the same amount of the drug was given and the movements reduced to five. The injections were continued every other day and a complete re-establishment of health resulted.

